# Oral administration of resveratrol or lactic acid bacterium improves lens elasticity

**DOI:** 10.1038/s41598-021-81748-w

**Published:** 2021-01-26

**Authors:** Hayato Nagashima, Nobunari Sasaki, Sachie Amano, Shigeru Nakamura, Motoshi Hayano, Kazuo Tsubota

**Affiliations:** 1 Department of Ophthalmology, Keio University School of Medicine, 35 Shinanomachi, Shinjuku-ku, Tokyo, 160-8582 Japan; 2 Department of Plastic and Reconstructive Surgery, Keio University School of Medicine, Tokyo, Japan; 3 Tsubota Laboratory, Inc., Tokyo, Japan

**Keywords:** Lens diseases, Ageing

## Abstract

A decrease in the elasticity of the ocular lens during aging is associated with loss of the accommodative ability of the eye, leading to presbyopia. Although near vision impairment is a social issue affecting the length of healthy life expectancy and productivity of elderly people, an effective treatment to improve near vision has not yet become available. Here we examined the effect of *Enterococcus faecium* WB2000, *Lactobacillus pentosus* TJ515, and resveratrol on lens elasticity in rats, where the stiffness of the ocular lens increases exponentially during the aging process. A combination of WB2000 and resveratrol improved lens elasticity not only in the long term but also with just short-term treatment. In addition, TJ515 decreased stiffness in the eye lens with long-term treatment. Therefore, the oral administration of WB2000 and resveratrol or TJ515 may be a potential approach for managing the progression of near vision impairment.

## Introduction

The accommodation ability to focus on near subjects is known to be decreased with age. The near vision impairment and presbyopia affect 1.8 billion people in 2015 (25% of people worldwide), with the number expects to reach 2.1 billion in 2030 given the expanding global population^1^. Although the near vision impairment has an impact on a healthy life expectancy and work productivity, current treatments remain limited to wearing glasses or contact lenses and surgical intraocular lens insertion^2^.

Lens elasticity is known to correlate with the near vision impairment in humans and the stiffness of the lens in the cortex and nuclear region is drastically increased with aging^3–6^. Pirenoxine eye drops prevented hardening of the lens in rats and Pirenoxine improved the accommodation ability in human in a clinical study^7^. Anti-oxidant supplementation and periocular warming were also observed to improve accommodation in human^8,9^. Lens fiber cells in the cortex and nuclear region are differentiated from lens epithelial cells accompanied by the loss of subcellular organelles including nuclear DNA in an autophagy-independent manner^10^. Given that protein synthesis and turnover are completely abolished in lens fiber cells, the maintenance of long-lived protein is crucial for continued lens homeostasis. Posttranslational modifications, such as disulfide bonding, deamidation, glycation, and oxidation, are increased with age, with the greater rate of disulfide bond formation in α-, β-, and γ-crystallin capable of inducing the appearance of high-molecular-weight crystalline^5,11–16^. Existing research suggests the administration of lipoic acid choline ester (LACE), derived from lipoic acid, reduced the concentration of disulfides in protein in mice, leading to the restoration of lens elasticity^17^. Some droplets including LACE are currently undergoing clinical trials to evaluate their efficacy and safety as the development of a new therapeutic agent to restore accommodative amplitude is of great interest^18^.

Resveratrol, a polyphenol found in grape or other plants, has shown an antioxidant capacity in neurodegenerative disease, the aging heart, and vascular diseases as well as the ability to facilitate lifespan elongation^19^. Nonspecific thiol oxidation induces disulfide binding, leading to protein aggregation. Antioxidative activity may play a crucial role in mitigating age-related ocular diseases such as glaucoma and age-related macular disease through the inhibition of reactive oxygen (ROS)^20–25^. However, though resveratrol is known to prevent oxidative stress in human lens epithelial cells, its effects on lens stiffness and the progression of the near vision impairment remain unknown.

Moreover, lactic acid bacteria, one of the groups of probiotic bacteria, have biological functions including antioxidative properties^26–29^. The long-term administration of a lactic acid bacterium suppressed retinal inflammation and retinal cell loss in aged mice^30^.

In the present study, we measured the stiffness of the rat lens to confirm that the elasticity of the lens is decreased during aging. Further, we evaluated the effects of the application of resveratrol and two lactic acid bacteria, WB2000 and TJ515, on the stiffness of the rat lens. Both the short- and long-term administration of resveratrol and WB2000 in combination mitigated the increase in stiffness of the rat lens typically experienced during aging, whereas the administration of TJ515 alone decreased the lens stiffness with long- but not short-term administration. These results indicate that the oral supplementation of an antioxidative diet could be a potential candidate to ameliorate the near vision impairment and presbyopia.

## Results

### Measurement of the lens elasticity of young and aged rats

**Figure 1:**
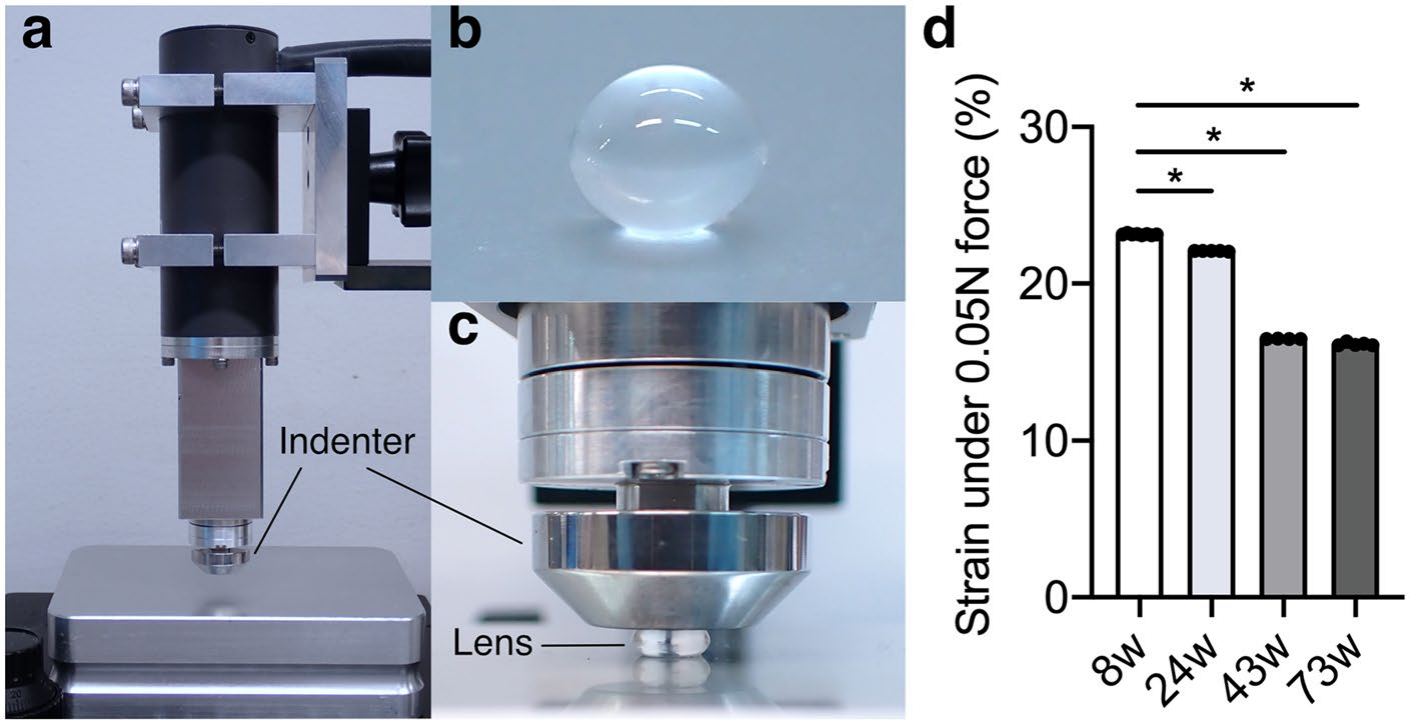
The measuring instrument, rat lens sample and the elasticity of young, middle and aged rat lenses. (**a**) The force–displacement measuring instrument with flat indentation. (**b**,**c**) The rat lens sample were put on a stage and a 12 mm-diameter flat indenter applied a load on the lens along the anteroposterior axis of the lens from above to measure the force and indentation displacement until 5.0 N (N) with the speed of 0.1 mm/s. (**d**) The lenses extracted from young (8-week-old, n = 8), middle-aged (24-week-old and 43-week-old) and old-aged (73-week-old, n = 5) male rats were assessed. The percentages of strain for the four age groups when lenses were pushed with 0.05 newtons (N) of force are shown in the bar graph. Data are presented as mean ± SEM. The asterisk indicates a statistically significant difference compared to 8-week-old (*P < 0.05, one-way analysis of variance followed by Tukey’s post-hoc test).

To compare the elasticity of the lens during aging, crystalline lenses were extracted from young (8-week-old), middle-aged (24-week-old and 43-week-old) and old-aged (73-week-old) male rats and their stiffness was measured using a force–displacement measuring instrument. Previously, a coverslip lens-squeezing method and a height gauge relying on an electronic balance method were used to measure the distance of strain when pushed with a specific force^17,31^. In this study, we quantified the stiffness of the lens at 0.05 Newtons (N) with the instrument (Fig. 1a–c). The group of aged rats showed decreased strain (%, the changes of lens anteroposterior diameter) relative to that in the group of young rats, indicating that the stiffness of the lens was increased in the former (Fig. 1d; Table 1). Changes in the elasticity of lens during aging are conserved across species ranging from mice to humans^3,5,31–33^.

**Table 1:** The elasticity of young, middle and aged rat lenses was measured with a force–displacement measuring instrument.

Age	Strain (%)	% of strain (compared to 8-week-old)	Sample number	*P* value (compared to 8-week-old)
8-week-old	23.14 ± 0.04	100	8	–
24-week-old	22.08 ± 0.02	98.5	5	< 0.0001*
43-week-old	16.47 ± 0.01	71.2	4	< 0.0001*
73-week-old	16.14 ± 0.10	69.7	5	< 0.0001*

As shown in Fig. 1d, the lenses extracted from young (8-week-old, n = 8), middle (24-week-old, n = 5 and 43-week-old, n = 4) and aged (73-week-old, n = 5) male rats were assessed. The percentages of strain for the four age groups when lenses were pushed with 0.05 newtons (N) of force are shown. Data are presented as mean ± SEM. The asterisk indicates a statistically significant difference compared to 8-week-old (*P < 0.05, one-way analysis of variance followed by Tukey’s post-hoc test).

### The long-term effects of resveratrol and lactic acid bacteria on the elasticity of the rat lens

**Figure 2:**
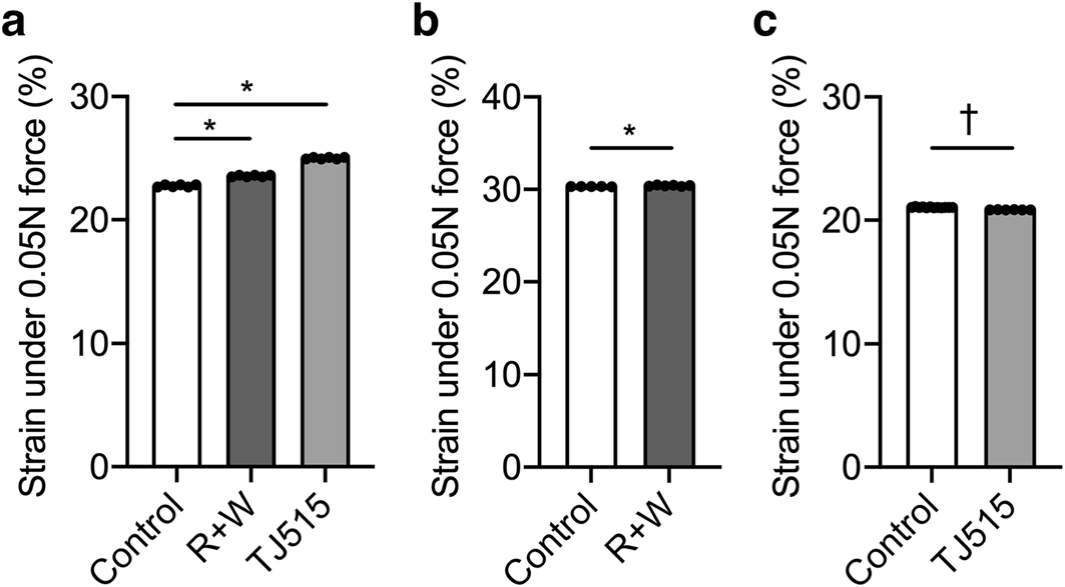
The effects of treatment with resveratrol and WB2000 together or TJ515 alone on rat lens elasticity. (**a**) Male rats (12-week-old) received the represented supplements alone or in combination by daily oral administration for 40 weeks. After the treatment period, rat lenses were extracted and their elasticity was measured (n = 6 in each group). The percentages of strain for the three groups (two treatment and one control) when the lenses were pushed with 0.05 Newtons (N) of force are shown in the bar graph. (**b**) The effects of short-term treatment with resveratrol and WB2000 on rat lens elasticity. Male rats (5-week-old) received resveratrol and WB2000 (R+W) in combination (n = 6) or the vehicle (control; n = 5) by daily oral administration for four weeks. After the treatment period, rat lenses were extracted and their elasticity was measured. The percentages of strain when the lenses were pushed with 0.05 newtons (N) of force are shown in the bar graph. (**c**) The effects of short-term treatment with TJ515 on rat lens elasticity. Male rats (5-week-old) received TJ515 (n = 6) or vehicle (control; n = 12) by daily oral administration for three weeks. After the treatment period, rat lenses were extracted and their elasticity was measured. The percentages of strain when the lenses were pushed with 0.05 newtons (N) of force are shown in the bar graph. Data are presented as mean ± SEM. The asterisk and dagger indicate statistically significant difference compared to each control (*, ^†^*P* < 0.05, one-way analysis of variance followed by Tukey’s post-hoc test or Student’s t-test). R + W = Resveratrol + WB2000.

To investigate the effects of resveratrol and lactic acid bacteria on rat lens elasticity, we treated male rats with resveratrol and the lactic acid bacterium WB2000 in combination or with another lactic acid bacterium, TJ515, alone by daily oral administration from 12 to 52 weeks of age. After 40 weeks of treatment, ocular lenses were extracted, then assessed for stiffness when pushed with 0.05 N of force (Fig. 2a). The combination of resveratrol and WB2000 yielded results of increased strain as compared with those of control vehicle-treated rat lenses (Fig. 2a; Table 2). Additionally, treatment with TJ515 alone for 40 weeks also increased the strain relative to that recorded in control vehicle-treated rats (Fig. 2a; Table 2). These results indicate that the elasticity of the rat lens is affected by the long-term oral administration of the combination of resveratrol and WB2000 or TJ515 alone.

**Table 2:** The effects of treatment with resveratrol and WB2000 together or TJ515 alone on rat lens elasticity.

Term	Period (week)	Supplements	Strain (%)	% of strain (compared to each control)	Sample number	*P* value (compared to each controls)
Long-term	40	Control	22.75 ± 0.08	100.00	6	–
		Resveratrol + WB2000	23.55 ± 0.07	103.52	6	< 0.0001*
		TJ515	25.00 ± 0.06	109.89	6	< 0.0001*
Short-term	4	Control	30.32 ± 0.01	100.00	5	–
		Resveratrol + WB2000	30.40 ± 0.06	100.26	6	< 0.0001*
Short-term	3	Control	21.04 ± 0.04	100.00	12	–
		TJ515	20.85 ± 0.01	99.10	6	< 0.0001^†^

As shown in Fig. 2, the percentages of strain for the three groups when lenses were pushed with 0.05 Newtons (N) of force are shown. Data are presented as mean ± SEM. The asterisk and dagger indicate statistically significant difference compared to each control (*, ^†^*P* < 0.05, one-way analysis of variance followed by Tukey’s post-hoc test or Student’s t-test).

### The short-term effects of resveratrol and lactic acid bacteria on the elasticity of the rat lens

Considering the improvements in rat lens elasticity gained with long-term treatment with TJ515 alone or the combination of resveratrol and WB2000, we next examined whether these supplements ameliorate the stiffness of the rat lens with only short-term treatment. At this stage of the research, male rats were treated with TJ515 or the combination of resveratrol and WB2000 by daily oral administration for 4 weeks instead of 40 weeks. The distance of strain in the group of rats treated with the combination of resveratrol and WB2000 was increased, indicating that the lens elasticity was improved by oral supplementation with resveratrol and WB2000 in a short period of time (Fig. 2b; Table 2). Meanwhile, rats treated with TJ515 alone for four weeks showed decreased lens strain as compared with that measured in control vehicle-treated rats (Fig. 2c; Table 2). These results together indicate that the combination of resveratrol and WB2000 possesses the potential to improve rat lens elasticity both in the short and long term, while TJ515 only functions as a long-term treatment to decrease the degree of lens stiffness in rats. To gain an understanding of the effects of resveratrol and lactic acid bacteria in the context of near vision and presbyopia, a clinical study assessing resveratrol, WB2000, and TJ515 is necessary in human.

## Discussion

Here, we showed that the stiffness of a crystalline lens in rats is increased with age and that daily oral administration of resveratrol and lactic acid bacteria increased the lens elasticity. In Helmholtz theory, short-range accommodation is associated with the degree of lens elasticity. Previously, we showed that lens elasticity was decreased in a smoking model in rats and pirenoxine eye drops, which have been used for the management of cataract, could improve the lens elasticity in rats^7^. Furthermore, the progression of near vision impairment was prevented by pirenoxine treatment in a clinical study^7^. Pirenoxine is known to maintain transparency in the crystalline lens by reducing the number of calcium ions, which have a crucial role in the aggregation of lens crystallin^34,35^. The homeostasis of lens proteins is associated with modifications including oxidation, deamidation, glycation, and the formation of disulfide bonds^36^. Indeed, in previous research, advanced glycation end-products mediated crosslinking and disulfide exchange among the proteins in the crystalline lens that are increased during aging^11,37^.

Resveratrol, contained in grapes and skin of peanuts, is used already as a dietary supplement and is known to have a beneficial effect on cancer, type 2 diabetes, and cardiovascular disease, owing to the activation of *SIRT1* and its protective antioxidant properties^38–40^. In addition, mixed dietary supplementation containing lactoferrin, fish oil, and *Enterococcus faecium* WB2000 decreased the amount of ROS production from the lacrimal gland in rats^41^. The oxidation of cysteine thiol by ROS is known to lead to the formation of protein disulfide bonds, which is a cause of protein aggregation^42,43^. The antioxidant response facilitated through redox-regulated proteins and the reduced form of glutathione possesses an ability to prevent protein disulfide bonding^44^. Therefore, the combination of resveratrol and WB2000 may serve as an antioxidant and could improve the elasticity of the crystalline lens. Although resveratrol has the antimicrobial activity against lactic acid bacteria, adding lactic acid bacteria concurrently may support the function of gut microbiota^45^. Separately, we observed that treatment with *Lactobacillus pentosus* TJ515 isolated from fermented food in Thailand increased the elasticity of the crystalline lens in rats, so dietary supplementation with TJ515 could also have a potential implication for the near vision impairment. Some types of lactic acid bacteria, *Lactobacilus* species are implicated in modulating immune response on autoimmune diseases such as rheumatoid arthritis, multiple sclerosis and inflammatory bowel disease^46–51^. Since aging accelerates constitutive low-grade inflammation and higher prevalence of autoimmunity even in the eye, the dietary supplement of TJ515 could also be a potential implication for the near vision^52,53^.

It is reported that symptoms of asthenopia including irritating and tired eyes, blurred vision, and a dry eye sensation, accompany the pre- and early stages of presbyopia^54^. Therefore, the treatment of presbyopia may ameliorate those symptoms of asthenopia. The pre- and early stages of presbyopia, those can be detected by the measurement of the DCNFVA (distance-corrected near functional visual acuity) begin at about 30 years old^54,55^. It is interesting that our short-term experiment exhibited ameliorated effects on the lens elasticity of younger rats (Fig. 2b,c). The profits of any investigation to treat the near vision impairment and presbyopia will not only improve health life expectancy but also work productivity, which will serve as checkpoints in efforts to meet sustainable development goals coordinated by the World Health Organization^56^.

## Methods

### Reagents

Resveratrol (purity > 99%, observed value) extracted from Indokinoki (*Pterocarpus marsupium*) was purchased from Vidya Japan (Tokyo, Japan). Lactic acid bacteria, WB2000, and TJ515 were provided by Wakamoto Pharmaceutical (Tokyo, Japan).

### Measurement of rat lens elasticity with a force–displacement measuring instrument with flat indentation

The elasticity of rat lenses was measured by using a force–displacement measuring instrument with flat indentation (YAWASA, Tec Gihan Co., Ltd., Tokyo, Japan). Briefly, lenses isolated from rats were immediately put on a metallic flat stage and the 12 mm-diameter flat indenter was positioned above the lens. Then the indenter gradually applied a load on the lens along the anteroposterior axis of the lens from above until 5.0 N (N) with the speed of 0.1 mm/s. The force and indentation displacement were recorded every 180^–1^ s to generate the force–displacement curve. We calculated the mean and SEM of the strain (%) under 0.05 N and assessed the difference statistically.

### Animals

We consulted the ARRIVE guideline (https://arriveguidelines.org/) to ensure proper reporting of animal experiments. The Keio University Institutional Animal Care and Use Committee approved all animal experiments (approval number: 17074), which were performed in Keio University School of Medicine, according to the Institutional Guidelines on Animal Experimentation at Keio University.

Male Wistar rats were purchased from Oriental Yeast (Tokyo, Japan). For the comparison of lens elasticity between young and aged rats, eight 8-week-old male rats, five 24-week-old male rats, four 43-week-old male rats and five 73-week-old male rats were prepared and each groups’ lenses were extracted to measure the elasticity using the YAWASA device.

For the evaluation of the effects of long-term treatment, 12-week-old male rats were divided into six groups and orally administrated the vehicle (n = 6), TJ515 (0.0070 mg daily) (n = 6), or a mixture of resveratrol (0.088 mg daily) and WB2000 (0.042 mg daily) (n = 6) for 40 weeks. After treatment, rat lenses were extracted to measure the elasticity using the YAWASA device.

For the evaluation of the effects of short-term treatment with the mixture of resveratrol and WB2000, 5-week-old male rats were divided into two groups and orally administrated the vehicle (n = 5) or a mixture of resveratrol (0.44 mg daily) and WB2000 (0.21 mg daily) (n = 6) for 4 weeks.

Finally, for the evaluation of the effects of short-term treatment with TJ515, 5-week-old male rats were divided into two groups and orally administrated either the vehicle (n = 12) or TJ515 (0.0070 mg daily) (n = 6) for 3 weeks. After treatment, rat lenses were extracted to measure the elasticity using the YAWASA device.

### Statistical analysis

Statistical analyses were performed using GraphPad Prism (GraphPad Software, San Diego, CA, USA). Data were expressed as mean ± standard error of the mean (SEM). The analysis of experimental data was performed using the two tailed Student’s *t*-test or one-way analysis of variance, followed by Tukey’s post-hoc test. A *P* value < 0.05 was considered to be statistically significant.
